# Methimazole Inhibits the Expression of GFAP and the Migration of Astrocyte in Scratched Wound Model *In Vitro*

**DOI:** 10.1155/2020/4027470

**Published:** 2020-04-13

**Authors:** Lina Zhao, Xianyu Zhang, Chunhai Zhang

**Affiliations:** ^1^Department of Thyroid Surgery, China-Japan Union Hospital of Jilin University, Changchun, Jilin, China; ^2^Department of Hand Surgery, China-Japan Union Hospital of Jilin University, Changchun, Jilin, China

## Abstract

Astrocytes respond to central nervous system (CNS) insults with varieties of changes, such as cellular hypertrophy, migration, proliferation, scar formation, and upregulation of glial fibrillary acidic protein (GFAP) expression. While scar formation plays a very important role in wound healing and prevents further bleeding by forming a physical barrier, it is also one of key features of CNS injury, resulting in glial scar formation (astrogliosis), which is closely related to treatment resistant epilepsy, chronic pain, and other devastating diseases. Therefore, slowing the astrocytic activation process may give a time window of axonal growth after the CNS injury. However, the underlying mechanism of astrocytic activation remains unclear, and there is no effective therapeutic strategy to attenuate the activation process. Here, we found that methimazole could effectively inhibit the GFAP expression in physiological and pathological conditions. Moreover, we scratched primary cultures of cerebral cortical astrocytes with and without methimazole pretreatment and investigated whether methimazole could slow the healing process in these cultures. We found that methimazole could inhibit the GFAP protein expression in scratched astrocytes and prolong the latency of wound healing in cultures. We also measured the phosphorylation of extracellular signal-regulated kinase (ERK) in these cultures and found that methimazole could significantly inhibit the scratch-induced GFAP upregulation. For the first time, our study demonstrated that methimazole might be a possible compound that could inhibit the astrocytic activation following CNS injury by reducing the ERK phosphorylation in astrocytes.

## 1. Introduction

Astrocytes are the most abundant cell type in the CNS, which can support the neighbouring neurons and integrate the nervous communication unit in the form of a “tripartite synapse” [1]. Astrocytes have critical roles in the maintenance of ion and neurotransmitter homeostasis [2]. In CNS, astrocytes undergo extensive physiological and morphological changes following insults, including trauma and surgery [3]. The changes in astrocytic morphology, protein expression, and hyperplasia are called as “reactive astrogliosis” [4]. The reactive astrocytes could facilitate the process of wound healing and might play a tissue-protective function [5]. However, astrogliosis could also become a detrimental process for the neuronal functional recovery if the cellular reaction occurs in a very fast way, forming a physical barrier that blocks the possible neuroregeneration [6]. Astrogliosis could also inhibit adaptive neural plasticity that is an essential process of neuronal recovery following injury [5]. A close association between astrogliosis and chronic CNS diseases, such as epilepsy, chronic pain, and brain trauma, has been established [7]. Therefore, identifying the possible compounds that could slow down the activation process of the astrocyte may open a new road for neuronal functional recovery and provide more information to preclinical studies, such as in animal models.

The increased expression of the GFAP protein, a key component of the cytoskeleton protein in astrocytes, is considered as a fundamental change of astrocytic activation [8]. Moreover, a study has suggested that upregulation of GFAP expression could be inhibited by an anti-inflammation compound, aspirin, via targeting on the NF-*κ*B signaling pathway [9]. Also, recent studies further indicated that GFAP upregulation and the above-mentioned other features of astrocyte activation are closely be influenced by the “secondary” neuroinflammation in the CNS after injury and *vice versa* [10]. Extracellular matrix molecules, such as chondroitin sulfate proteoglycans (CSPGs), are essential components of glial scars [10]. In particular, three core proteins play very important roles in the formation of the glial scar [11–13].

Therefore, regulating the GFAP and CSPG core protein expression and inflammatory response following CNS injury could be a potential useful strategy to attenuate the astrogliosis process and scar formation.

Methimazole is an antithyroid compound that has been broadly used in the treatment of hyperthyroidism and Graves disease via preventing the production of the level of thyroid hormones from the thyroid gland. Interestingly, methimazole was also found to have significant impact on CNS by influencing the levels of varieties of neurotransmitters [14]. A previous study also suggested that methimazole could regulate the opioid receptor expression in the brain [15]. Recently, one study demonstrated that methimazole could attenuate glial cell function in the CNS of diabetic mice [16]. However, whether methimazole could directly affect the activation of the astrocyte under injury in a short period of time remains elusive. The anti-inflammation effect of methimazole has been established in literature [17].

In the present study, we investigate whether methimazole could exert direct effect on the activation process of the astrocyte in an *in vitro* scratch wound model by exploring the effects of methimazole on the GFAP protein expression and wound healing. We also explored the possible mechanisms by which methimazole regulated the cellular and molecular responses of astrocytes.

## 2. Materials and Methods

### 2.1. Cells and Treatments

Astrocytes were set up by using one-day-old newborn ICR mice as previously reported with minor modifications [18]. Cortical explants were isolated and cut into a 1 mm cube. After being mechanically vortexed, cells were plated on 35 mm dishes at the density of 1 × 10^5^ per dish. These astrocytic cultures were maintained on Dulbecco's Modified Eagle's Medium (DMEM, Life Technologies) with 10% fetal bovine serum (FBS, Life Technologies) for the first two weeks and changed to 7% for the next two weeks. The medium was changed every 3 days. Astrocytes were used for experiments when they were 4 weeks old, which were considered as functionally mature [18]. Methimazole was purchased from Sigma and dissolved in DMEM. The doses used in the study were referred from the previous publication [19]. Animal protocols were approved by the Experimental Animal Ethics Committee of China-Japan Union Hospital, Jilin University, Jilin Province, China.

### 2.2. Cell Viability Assay (MTT)

We measured the cell viability with MTT assay as described by another group previously [20]. Briefly, astrocytes for MTT were plated in 96-well plates instead of culture dishes. 4-week-old astrocytes in the plates were used and treated with different doses of methimazole for 12 hours. At the end of each experiment, we applied MTT solution directly into the well medium (10%), and then the plates were incubated for 3-4 hours at 37°C. Optical densities were probed at 570 nm, and viabilities were expressed as a relative percentage compared to their controls.

### 2.3. Scratch Wound Assay

Scratch wound assay was used to assess the effects of methimazole on the wound healing in the scratched cultured astrocytes. Cells used for scratch were maintained in 35 mm dishes until 4 weeks old. The scratch wound was then created by using plastic pipette tips (10–20 *μ*L pipette tips) as previously reported by [6]. We washed the dishes immediately after the scratch to remove dead cells and other debris generated during the scratch. The scratched dishes were then treated with or without methimazole. During this time, the wound gaps between groups were repeatedly imaged to measure the distance between two edges of the wounds in each dish.

### 2.4. Western Blot

To measure the expression levels of proteins, the total proteins were extracted from the cultured astrocytes. The cold lysis buffer contained a phosphatase and protease inhibitor cocktail (Sigma) to minimize the protein degradation. The samples containing total proteins were denatured with boiled water for 5 minutes and then separated on 12% SDS-PAGE with electrophoresis. The proteins were transferred onto nitrocellulose membranes followed by blocking for 2 hours with BSA or skim milk. Primary antibodies of GFAP (1 : 1000, Santa Cruz), p-ERK (1 : 1000, Cell Signaling), ERK (1 : 1000, Cell Signaling), p-JNK (1 : 1000, Cell Signaling), and JNK (1 : 1000, Cell Signaling) and the corresponding secondary antibodies were used here. *β*-Actin (Santa Cruz) was used as an internal control. Quantitative results of the protein expression levels were presented as a ratio of these proteins compared to the *β*-actin accordingly.

### 2.5. ELISA

The secreted levels of IL-1*β* and IL-6 were probed in the extracted proteins of astrocytes with an ELISA kit according to the protocols in the manual (eBiosciences). Generally, we collected the cell-free medium from cultures with or without treatment. And then the levels of IL-1*β* and IL-6 were measured using the ELISA assay kit accordingly. Each sample was duplicated for the assay, and averaged values were used for analysis. Total protein concentration in each sample was measured with a BCA assay (Abcam), and values of IL-1*β* and IL-6 were presented as the ratio of them compared to total protein.

### 2.6. Quantitative Real-Time PCR

Total RNA was harvested from cultured astrocytes with the RNA Mini-Preps Kit (Sangon Biotech, Shanghai) and followed by reverse transcription to cDNA and quantitative PCR in the CFX96 Real-Time System (Bio-Rad). Primers used here were adapted from a previous publication and GAPDH was used as the internal control [21, 22] (Table 1). Values of all genes were normalized to GAPDH, and quantification was calculated by using the delta delta threshold cycle method as previously reported [18].

### 2.7. Statistical Analysis

All data were expressed as mean ± S.E.M. in the study. Statistical analysis was carried out with the software Prism. One-way ANOVA followed by Newman–Keuls post hoc test or *t*-test was used for the group analysis. Differences were considered statistically significant when *p* < 0.05.

## 3. Results

### 3.1. Effects of Methimazole on the Cell Viability, GFAP, and CSPG Core Protein Expression in Scratched Astrocytes

**Figure 1:**
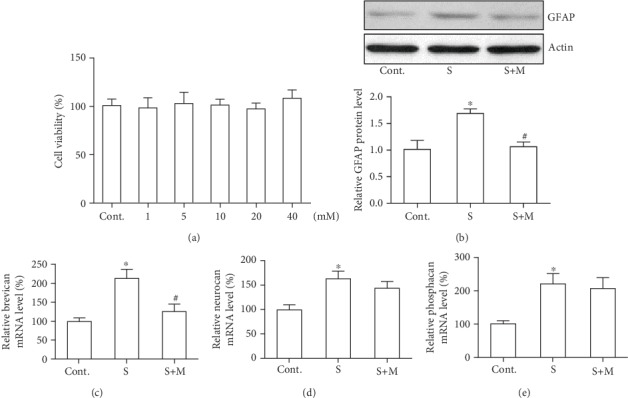
Methimazole inhibited the upregulation of GFAP protein and brevican mRNA expression in scratched astrocytes (12 hours). (a) Methimazole exerted no cell toxic effect on astrocyte viability study. (b) Methimazole inhibited GFAP upregulation in scratched astrocytes. (c) Methimazole inhibited brevican mRNA expression in scratched astrocytes. (d) Methimazole did not inhibit neurocan mRNA expression in scratched astrocytes. (e) Methimazole did not inhibit phosphacan mRNA expression in scratched astrocytes. All values were expressed as mean ± SEM, *n* = 10 (a) or 5 (b–e). ^∗^*p* < 0.05 vs. control; ^#^*p* < 0.05 vs. scratch.

First, the effects of methimazole on the cell viability of the astrocyte were investigated. We treated astrocytes in culture with different doses of methimazole (1, 5, 10, 20, and 40 mM). And we found there were no obvious toxic effects of methimazole on the astrocytes (Figure 1(a)). Thereafter, 5 mM was used for the following study in the scratch wound model based on the cell viability result and a previous report on a cell culture model [19]. Next, we tested our primary hypothesis whether methimazole could inhibit the upregulation of the GFAP protein in a scratched astrocyte culture. As shown in Figure 1(b), scratch injury induced a significant increased expression of the GFAP protein after 12 hours, which is consistent with a previous report [6]. However, the increase of the GFAP protein level could be inhibited by the pretreatment of methimazole in the cultured astrocytes. With the use of RT-PCR, we measured the relative expression levels of three core CGPG proteins (neurocan, brevican, and phosphacan) in the scratched astrocytes to see whether methimazole could affect the expression of their mRNA expression levels. As shown in Figures 1(c)–1(e), the scratch caused increased expression of the brevican mRNA level but not neurocan and phosphacan, although there was a trend of increased expression of these two genes. Interestingly, methimazole could prevent the increased expression of brevican.

### 3.2. Effects of Methimazole on the ERK and JNK Protein Expression in Scratched Astrocytes

**Figure 2:**
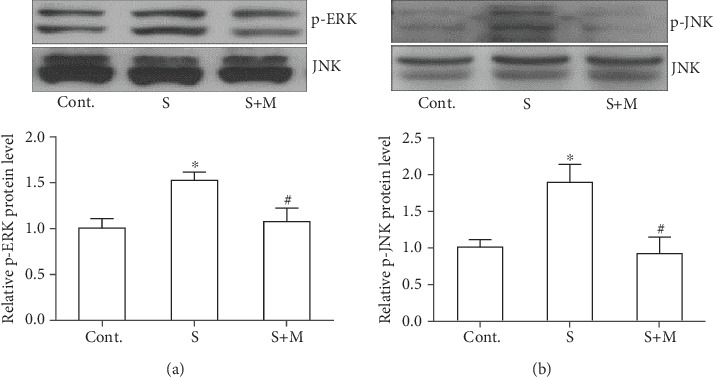
Methimazole attenuated the increased expression of phosphorylated JNK and ERK proteins (30 minutes). (a) Methimazole prevented the increased expression of p-ERK in scratched astrocytes. (b) Methimazole prevented the increased expression of p-JNK in scratched astrocytes. All values were expressed as mean ± SEM, *n* = 5. ^∗^*p* < 0.05 vs. control; ^#^*p* < 0.05 vs. scratch.

Next, we also explored the possible mechanism by which methimazole could regulate the GFAP protein expression and the astrocytic activation process in the scratch wound. Studies have validated that ERK and JNK phosphorylation actively participated in the astrocytic activation [6, 23]. Therefore, we performed western blot to investigate the expression level of ERK and its phosphorylated form in these cultured astrocytes. We found that the scratch induced significant upregulated expression of phosphorylated ERK, which could be prevented by the pretreatment of methimazole (Figure 2(a)). We also explored the expression level of phosphorylated JNK and found that phosphorylated JNK was significantly increased at 30 minutes after the scratch in cultured astrocytes, which could be effectively reduced by the pretreatment of methimazole too (Figure 2(b)). The increased expression of phosphorylated JNK and ERK was in line with the results from aforementioned publications. And our results implied that methimazole might inhibit the astrocytic activation in the scratch wound model by preventing the phosphorylation of JNK and ERK.

### 3.3. Effects of Methimazole on the Cell Migration in Scratched Astrocytes

**Figure 3:**
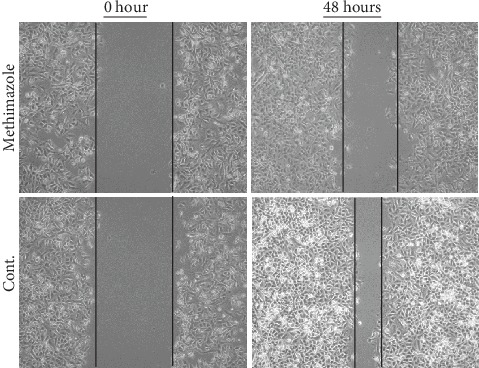
Methimazole decreased the capability of migration of astrocytes in the scratch wound model (48 hours).

Then, we tested whether methimazole could inhibit the migration of reactive astrocytes that are along the wound edge. Increased capacity of migration in reactivated astrocytes has been reported previously [24]. We treated the cultured astrocytes subjected to scratch injury with methimazole and then took images with a microscope to monitor the velocity of wound healing in the dishes among different groups. We found that the process of wound healing was initiated very quickly after the scratch as shown in the images, in which astrocytes protruded processes to cover the wound. However, the gap closure was much slower in astrocytes treated with methimazole than in the astrocytes without treatment (Figure 3). These results implied that methimazole could decrease the migration of scratched astrocytes, which might be the result from the inhibition on astrocytic activation.

### 3.4. Effects of Methimazole on the Protein Expression of IL-1*β* and IL-6 in Scratched Astrocytes

**Figure 4:**
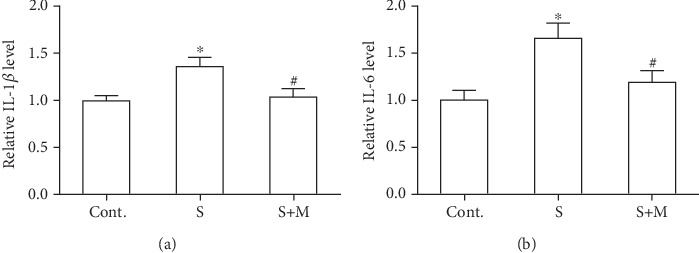
Methimazole prevented the increased release of IL-1*β* and IL-6 cytokines in the culture medium of scratched astrocytes. (a) Methimazole prevented the increased expression of IL-1*β* in scratched astrocytes. (b) Methimazole prevented the increased expression of IL-6 in scratched astrocytes. All values were expressed as mean ± SEM, *n* = 8. ^∗^*p* < 0.05 vs. control; ^#^*p* < 0.05 vs. scratch.

Based on the above findings, we hypothesized that methimazole could be a promising compound to slow down the astrocytic reactivation process following CNS injuries as we expected. To further confirm the assumption, we also investigated whether methimazole could influence the inflammatory response of astrocytes in the scratched cultures. We performed ELISA to measure the levels of cytokines, IL-1*β*, and IL-6 in scratched astrocytes, both of which are well-known cytokines that could be upregulated in scratched astrocytes [24]. As shown in Figures 4(a) and 4(b), we found that the scratch injury caused significant increased expression of these two cytokines, which suggested that scratch injury-induced astrocyte activation might lead to “hyperfunctional” astrocytes with the capability of boosting the products of these cytokines. Importantly, when pretreated with methimazole, the increased cytokine levels could be effectively reduced in the culture medium of astrocytes. These above results consistently implicated that methimazole could effectively attenuate the astrocyte-derived inflammatory response in the scratch wound culture model by inhibiting the astrocyte reactivation.

## 4. Discussion

Upregulation of the GFAP protein expression level is the one of the prominent features in astrogliosis [25]. And astrogliosis is considered to inhibit the neuronal repair after CNS injury by the rapid formation of a physical barrier [6]. Our study revealed that the antithyroid compound, methimazole, might be a promising compound that could facilitate nerve regeneration after CNS injury under some circumstances since it showed some beneficial effects on the astrocyte reactivation process in a scratch wound model of cultured astrocytes. Although astrocytic activation is an essential part of the CNS response following varieties of insults, it could exert adverse influence on the neuronal cell recovery [26]. Assumption has been established for decades that the physical barrier resulted from the astrogliosis being able to intervene in the process of nerve regeneration. Therefore, the findings here might identify a new available compound with an approved safety profile to modulate the robust and fast astrocytic activation after traumatic or surgery insults on CNS. We explored the possible effects of methimazole on the CSPG core proteins and found that methimazole could inhibit the scratch-induced upregulation of brevican. However, we could not detect significant increased expression of neurocan and phosphacan in our cultured astrocytes suffering scratch wound injury as the previous results in an animal model. This may be due to the expressing profiles of CSPG core proteins in cultured astrocytes not being identical with those in the animal model. Further study concerning the expression pattern of these proteins is warranted in the future.

Methimazole may act through different pathways to influence astrocytic function in CNS. First, it could prevent the reactivation of astrocytes, which was validated in our results by showing that methimazole inhibited the migration of astrocytes along the edges of the scratch wound. Secondly, methimazole could affect the function of astrocytes after being reactivated by insults, including the production of cytokines [27]. As shown in Figure 4, we found that methimazole significantly decreased the protein levels of IL-1*β* and IL-6 in the culture medium of scratched astrocytes. The capability of methimazole to attenuate the direct inflammatory response suggested an anti-inflammatory role. As mentioned above, methimazole has been found to inhibit the glial cell function in a diabetic mouse model [14]. Our results were consistent with these previous results. Moreover, neuroinflammation and cytokines could greatly impact the neuronal function via adversely regulating the synaptic plasticity or synaptic protein function [28, 29]. Therefore, the fact that methimazole could directly block the release of these cytokines after CNS injury could protect neurons from the “secondary attack” of the subsequent inflammatory response.

The beneficial effects of methimazole on astrocytic activation following injury were further confirmed by the results on the signaling pathway in our study. It has been well established that the MAPK pathway is closely associated with the reactivation process of astrocytes in injury, including the scratch wound [6]. We investigated the phosphorylation status of JNK and ERK among those MAPK-related proteins. Western blot results in our study demonstrated a significant increase of phosphorylated JNK and ERK. Importantly, the expression levels of these phosphorylated proteins in astrocytes after the scratch could be prevented by the pretreatment of methimazole.

There are varieties of advantages of methimazole for its possible application in CNS for the future. First, methimazole has been commercially available for many years in clinical medicine. Secondly, methimazole possesses the relative safety profile that could allow it to be used for a longer time for neuronal regeneration that usually takes months, even years. Third, study in animals as mentioned above has suggested the ability of methimazole on CNS infiltration. However, there are limitations to this present study. Our study is the first step to explore the possible application in an *in vitro* model. The findings and conclusion from this study are preliminary.

## 5. Conclusion

The present study provided *in vitro* data for the first time that methimazole possesses the potential to be used for the treatment of CNS injury by targeting astrocyte function. While more *in vivo* data is warranted in animal models, such as the stab wound model, the current finding may provide the preliminary evidence for a new application of the widely used compound in CNS injury by slowing down the activation process of astrocytes and allowing injured neurons to recover.

## Figures and Tables

**Table 1:** 

Genes	Forward	Reverse
Neurocan	GAGAGAGATTGCAGGCGCCGAGCTG	CTCGGTGCTGGGAGAATCCTTCATC
Phosphacan	TTGGCTCCTACTATCAACATCCTCC	AGCTCATCCCTCTCAGCAGCTGAAG
Brevican	CTCGATTTCGTTAGGGGACA	ATTTCCACCTGGTTTTGCTG
GAPDH	AACTTTGGCATTGTGGAAGG	GGATGCAGGGATGATGTTCT

## Data Availability

The data used to support the findings of this study are available from the corresponding author upon request.

## References

[1] Qiao J., Wang J., Wang H. (2016). Regulation of astrocyte pathology by fluoxetine prevents the deterioration of Alzheimer phenotypes in an APP/PS1 mouse model. *Glia*.

[2] Robel S., Buckingham S. C., Boni J. L. (2015). Reactive astrogliosis causes the development of spontaneous seizures. *The Journal of Neuroscience*.

[3] Sun D., Jakobs T. C. (2012). Structural remodeling of astrocytes in the injured CNS. *The Neuroscientist*.

[4] Sofroniew M. V., Vinters H. V. (2010). Astrocytes: biology and pathology. *Acta Neuropathologica*.

[5] Faulkner J. R., Herrmann J. E., Woo M. J., Tansey K. E., Doan N. B., Sofroniew M. V. (2004). Reactive astrocytes protect tissue and preserve function after spinal cord injury. *The Journal of Neuroscience*.

[6] Gao K., Wang C. R., Jiang F. (2013). Traumatic scratch injury in astrocytes triggers calcium influx to activate the JNK/c-Jun/AP-1 pathway and switch on GFAP expression. *Glia*.

[7] Pekny M., Pekna M. (2014). Astrocyte reactivity and reactive astrogliosis: costs and benefits. *Physiological Reviews*.

[8] Brahmachari S., Fung Y. K., Pahan K. (2006). Induction of glial fibrillary acidic protein expression in astrocytes by nitric oxide. *The Journal of Neuroscience*.

[9] Bae M.-K., Kim S.-R., Lee H.-J. (2006). Aspirin-induced blockade of NF-*κ*B activity restrains up-regulation of glial fibrillary acidic protein in human astroglial cells. *Biochim Biophys Acta*.

[10] Pekny M., Pekna M. (2016). Reactive gliosis in the pathogenesis of CNS diseases. *Biochimica et Biophysica Acta*.

[11] Sherman L. S., Back S. A. (2008). A 'GAG' reflex prevents repair of the damaged CNS. *Trends in Neurosciences*.

[12] Davies J. E., Tang X., Denning J. W., Archibald S. J., Davies S. J. (2004). Decorin suppresses neurocan, brevican, phosphacan and NG2 expression and promotes axon growth across adult rat spinal cord injuries. *The European Journal of Neuroscience*.

[13] Davies S. J., Fitch M. T., Memberg S. P., Hall A. K., Raisman G., Silver J. (1997). Regeneration of adult axons in white matter tracts of the central nervous system. *Nature*.

[14] Hashizume Y., Yamaki T., Hidaka H. (1977). Effect of anti-thyroid agents, methimazole and propylthiouracil, on brain noradrenaline content. *British Journal of Pharmacology*.

[15] Bhargava H. N., Ramarao P., Gulati A. (1988). Effect of methimazole-induced hypothyroidism on multiple opioid receptors in rat brain regions. *Pharmacology*.

[16] Nam S. M., Kim Y. N., Yoo D. Y. (2013). Hypothyroidism affects astrocyte and microglial morphology in type 2 diabetes. *Neural Regeneration Research*.

[17] Lagorce J. F., Moulard T., Rousseau A., Comby F., Buxeraud J., Raby C. (1997). Anti-inflammatory action of methimazole. *Pharmacology*.

[18] Wang J., Zhu S., Wang H. (2014). Astrocyte-dependent protective effect of quetiapine on GABAergic neuron is associated with the prevention of anxiety-like behaviors in aging mice after long-term treatment. *Journal of Neurochemistry*.

[19] Sue M., Akama T., Kawashima A. (2012). Propylthiouracil increases sodium/iodide symporter gene expression and iodide uptake in rat thyroid cells in the absence of TSH. *Thyroid*.

[20] Aldasoro M., Guerra-Ojeda S., Aguirre-Rueda D. (2016). Effects of ranolazine on astrocytes and neurons in primary culture. *PLoS One*.

[21] Susarla B. T., Laing E. D., Yu P., Katagiri Y., Geller H. M., Symes A. J. (2011). Smad proteins differentially regulate transforming growth factor-*β*-mediated induction of chondroitin sulfate proteoglycans. *Journal of Neurochemistry*.

[22] Thomas K. C., Zheng X. F., Garces Suarez F. (2014). Evidence based selection of commonly used RT-qPCR reference genes for the analysis of mouse skeletal muscle. *PLoS One*.

[23] Li D., Liu N., Zhao H. H. (2017). Interactions between Sirt 1 and MAPKs regulate astrocyte activation induced by brain injury in vitro and in vivo. *Journal of Neuroinflammation*.

[24] Lau L. T., Yu A. C. (2001). Astrocytes produce and release interleukin-1, interleukin-6, tumor necrosis factor alpha and interferon-gamma following traumatic and metabolic injury. *Journal of Neurotrauma*.

[25] Zhang D., Hu X., Qian L., O'Callaghan J. P., Hong J. S. (2010). Astrogliosis in CNS pathologies: is there a role for microglia?. *Molecular Neurobiology*.

[26] Li Y., Xu X.-L., Zhao D. (2015). TLR3 ligand poly IC attenuates reactive astrogliosis and improves recovery of rats after focal cerebral ischemia. *CNS Neuroscience & Therapeutics*.

[27] Filous A. R., Silver J. (2016). Targeting astrocytes in CNS injury and disease: a translational research approach. *Progress in Neurobiology*.

[28] Pendyala G., Chou S., Jung Y. (2017). Maternal immune activation causes behavioral impairments and altered cerebellar cytokine and synaptic protein expression. *Neuropsychopharmacology*.

[29] Werneburg S., Feinberg P. A., Johnson K. M., Schafer D. P. (2017). A microglia-cytokine axis to modulate synaptic connectivity and function. *Current Opinion in Neurobiology*.

